# Peripheral chemosensitivity is not blunted during 2 h of thermoneutral head out water immersion in healthy men and women

**DOI:** 10.14814/phy2.13472

**Published:** 2017-10-19

**Authors:** James R. Sackett, Zachary J. Schlader, Suman Sarker, Christopher L. Chapman, Blair D. Johnson

**Affiliations:** ^1^ Center for Research and Education in Special Environments Department of Exercise and Nutrition Sciences University at Buffalo Buffalo New York

**Keywords:** CO_2_ retention, ventilation, heart rate, blood pressure

## Abstract

Carbon dioxide (CO_2_) retention occurs during water immersion, but it is not known if peripheral chemosensitivity is altered during water immersion, which could contribute to CO_2_ retention. We tested the hypothesis that peripheral chemosensitivity to hypercapnia and hypoxia is blunted during 2 h of thermoneutral head out water immersion (HOWI) in healthy young adults. Peripheral chemosensitivity was assessed by the ventilatory, heart rate, and blood pressure responses to hypercapnia and hypoxia at baseline, 10, 60, 120 min, and post HOWI and a time‐control visit (control). Subjects inhaled 1 breath of 13% CO_2_, 21% O_2_, and 66% N_2_ to test peripheral chemosensitivity to hypercapnia and 2–6 breaths of 100% N_2_ to test peripheral chemosensitivity to hypoxia. Each gas was administered four separate times at each time point. Partial pressure of end‐tidal CO_2_ (PETCO_2_), arterial oxygen saturation (SpO_2_), ventilation, heart rate, and blood pressure were recorded continuously. Ventilation was higher during HOWI versus control at post (*P *= 0.037). PETCO_2_ was higher during HOWI versus control at 10 min (46 ± 2 vs. 44 ± 2 mmHg), 60 min (46 ± 2 vs. 44 ± 2 mmHg), and 120 min (46 ± 3 vs. 43 ± 3 mmHg) (all *P* < 0.001). Ventilatory (*P* = 0.898), heart rate (*P* = 0.760), and blood pressure (*P* = 0.092) responses to hypercapnia were not different during HOWI versus control at any time point. Ventilatory (*P* = 0.714), heart rate (*P* = 0.258), and blood pressure (*P* = 0.051) responses to hypoxia were not different during HOWI versus control at any time point. These data indicate that CO_2_ retention occurs during thermoneutral HOWI despite no changes in peripheral chemosensitivity.

## Introduction

Carbon dioxide (CO_2_) retention occurs during water immersion (Lambertsen et al. 1959; Jarrett 1966; Lollgen et al. 1976; Kerem et al. 1980, 1995; Salzano et al. 1984; Warkander et al. 1990; Lanphier and Bookspan 1999; Pendergast and Lundgren 2009; Pendergast et al. 2015). Increases in arterial CO_2_ content occur at rest and during exercise at various depths, gas concentrations, and breathing resistances (Salzano et al. 1984; Mummery et al. 2003; Cherry et al. 2009). CO_2_ retention increases the risk of CO_2_ toxicity (i.e., CO_2_ narcosis) during underwater excursions (Warkander et al. 1990; Lanphier and Bookspan 1999); therefore, determining underlying mechanisms that contribute to CO_2_ retention is important for divers. During water immersion, the thoracic cavity is subjected to an elevated hydrostatic pressure from the water column that causes high external breathing resistance (i.e., static lung load), which can contribute to respiratory muscle fatigue (Pendergast and Lundgren 2009; Pendergast et al. 2015). Furthermore, water immersion alters hemodynamics to increase central blood volume (Arborelius et al. 1972; Begin et al. 1976; Farhi and Linnarsson 1977; Bonde‐Petersen et al. 1992; Pendergast et al. 2015). Central hypervolemia during water immersion elevates the diaphragm and decreases lung compliance (Agostoni et al. 1966; Arborelius et al. 1972; Mummery et al. 2003; Cherry et al. 2009; Moon et al. 2009), which may increase dead space ventilation and reduce alveolar ventilation. The increased hydrostatic load on the chest wall and central hypervolemia appear to favor alveolar hypoventilation (Salzano et al. 1970; Thalmann et al. 1979; Hickey et al. 1987; Norfleet et al. 1987; Warkander et al. 1990; Lanphier and Bookspan 1999). Thus, alveolar hypoventilation might contribute to CO_2_ retention during water immersion (Cherry et al. 2009; Pendergast et al. 2015), and increase the risk for CO_2_ toxicity (Warkander et al. 1990; Lanphier and Bookspan 1999). However, the chemical control of ventilation is less understood during water immersion (Moon et al. 2009).

The chemical control of ventilation in humans is tightly regulated by the central and peripheral chemoreceptors which detect changes in arterial blood gases and pH (Kara et al. 2003). Chang and Lundgren (1995) have shown that central chemosensitivity is not altered during 10 min of water immersion, which indicates that the central chemoreceptors are not affected by brief thermoneutral water immersion. The peripheral chemoreceptors, comprised of the aortic and carotid bodies, are the primary oxygen sensors in the body (Kara et al. 2003; Prabhakar and Peng 2004). In addition to oxygen sensing (Kara et al. 2003; Prabhakar and Peng 2004), the peripheral chemoreceptors are activated when exposed to acute hypercapnia and contribute to the acute hypercapnic ventilatory response (Kara et al. 2003). In fact, the peripheral chemoreceptors account for approximately 35% of the increase in ventilation during acute hypercapnia (Smith et al. 2006; Wilson and Teppema 2016). Therefore, a reduction in peripheral chemosensitivity could contribute to CO_2_ retention during water immersion.

A possible mechanism which could contribute to the reduction in the chemical control of ventilation during water immersion is the interaction between the arterial baroreceptors and the peripheral chemoreceptors (Heistad et al. 1975; Koehle et al. 2010). Peripheral chemosensitivity is blunted during baroreceptor loading (Heistad et al. 1975); therefore, central hypervolemia during water immersion (Arborelius et al. 1972; Pendergast et al. 2015) could blunt peripheral chemosensitivity and play a role in CO_2_ retention. The purpose of our study is to test the hypothesis that peripheral chemosensitivity is blunted during HOWI in humans.

## Methods

## Subjects

Ten subjects (age: 23 ± 2 years, BMI: 26 ± 2 kg/m^2^, 3 women) participated in four visits: a screening visit, a familiarization visit, and two randomized experimental visits. Subjects self‐reported to be active, nonsmokers, not taking medications, and free from any known cardiovascular, metabolic, neurological, or psychological disease. Women were not pregnant, confirmed via a urine pregnancy test prior to the familiarization and experimental visits, and were tested during the first 10 days following self‐identified menstruation to control for menstrual cycle hormones (Minson et al. 2000). Each subject was informed of the experimental procedures and possible risks before giving informed, written consent. During the familiarization visit, all subjects were acquainted with the breathing apparatus (i.e., the mouthpiece and pneumatic switching valve) and gases that would be used during the experimental visits. The study was approved by the Institutional Review Board at the University at Buffalo, and performed in accordance with the standards set forth by the latest revision of the Declaration of Helsinki.

### Instrumentation and measurements

Height and weight were measured with a stadiometer and scale (Sartorius Corp., Bohemia, NY). Urine‐specific gravity was measured using a refractometer (Atago USA, Inc., Bellevue, WA). The partial pressure of end‐tidal carbon dioxide (PETCO_2_) was measured using a capnograph (Nonin Medical, Inc., Plymouth,). Since PETCO_2_ reflects PaCO_2_ throughout a wide range of physiological dead space (McSwain et al. 2010), including water immersion (Salzano et al. 1984; Mummery et al. 2003; Cherry et al. 2009), PETCO_2_ was used as a marker of PaCO_2_ in our study. Arterial oxygen saturation (SpO_2_) was measured using a finger pulse oximeter (Nonin Medical, Inc.) and beat to beat blood pressure was measured via the Penaz method (ccNexfin Bmeye NA, St. Louis, MO) on a hand that was supported above the water during HOWI. Blood pressure was corrected to heart level using a height correction sensor (ccNexfin Bmeye NA). Heart rate was measured continuously from a three lead ECG (DA100C; Biopac Systems, Inc., Goleta, CA). Inspired and expired ventilation were measured continuously using nonheated and heated pneumotachometers, respectively, (Hans Rudolph, Inc., Shawnee, KS) that were attached to a two‐way nonrebreathing valve and mouthpiece (Hans Rudolph, Inc.). Hemodynamic data were obtained at 500 Hz and ventilation data were captured at 62 Hz by a data acquisition system (Biopac MP 150, Goleta, CA) and stored on a personal computer for offline analyses. Minute ventilation, tidal volume, and respiratory rate were determined using the breath by breath respiratory analysis program of the data acquisition system (AcqKnowledge 4.2, Goleta, CA) by a blinded researcher. Abhorrent breaths (e.g., sigh, breath hold, etc.) were excluded and ventilation data are presented in BTPS. The rate of CO_2_ production (VCO_2_) was calculated as mean expired CO_2_ partial pressure (i.e., derived from the CO_2_ waveform) divided by barometric pressure minus water vapor pressure of the body (Siobal et al. 2013). Alveolar ventilation was calculated as the product of VCO_2_ and 863 divided by PETCO_2_ (West 2012). Dead space ventilation was calculated as minute ventilation minus alveolar ventilation. Stroke volume was determined via the arterial pressure waveform using Modelflow (ccNexfin Bmeye NA) and cardiac output was calculated as the product of heart rate and stroke volume. Total peripheral resistance was calculated as mean arterial pressure divided by cardiac output. The ratio of alveolar ventilation to cardiac output was calculated as an index of the ratio of alveolar ventilation to pulmonary perfusion (Derion et al. 1992; Levitzky 2013).

### Experimental approach

Subjects reported to the laboratory for two randomized experimental visits: (1) a HOWI visit and (2) a time‐control dry visit (control). Subjects arrived at the laboratory having refrained from strenuous exercise, alcohol, and caffeine for 12 h, and food for 2 h for both visits. Subjects also arrived to the laboratory euhydrated for both HOWI and control visits (urine‐specific gravity: 1.012 ± 0.007 and 1.015 ± 0.006, respectively). Subjects assumed a seated position for instrumentation in a temperature‐controlled laboratory (25 ± 2°C, 49 ± 8% relative humidity). Following at least 10 min of seated rest, baseline peripheral chemosensitivity to hypercapnia and hypoxia were measured. It has been suggested that peripheral chemosensitivity to both acute hypercapnia and hypoxia should be used in order to completely assess the peripheral chemoreflex (Chua and Coats 1995). Upon the completion of baseline measurements, the subjects either entered a pool (HOWI) or continued seated rest (control) for 2 h. HOWI consisted of seated rest in thermoneutral water (35.1 ± 0.2°C) up to the suprasternal notch. Over the next 2 h, peripheral chemosensitivity to hypercapnia and hypoxia were measured at 10, 60, and 120 min. Then, subjects exited the pool (HOWI) or remained seated (control), and peripheral chemosensitivity to hypercapnia and hypoxia were measured after 10 min of seated rest (i.e., post). During the peripheral chemosensitivity to hypercapnia and hypoxia tests, subjects were encouraged to breathe spontaneously as they viewed a nonstimulating documentary.

### Peripheral chemosensitivity to hypercapnia

Peripheral chemosensitivity to hypercapnia was measured via four carbon dioxide administrations (i.e., 13% CO_2_, 21% O_2_, and 66% N_2_) separated by 3 min of room air breathing. Briefly, using a pneumatic switching valve (Hans Rudolph, Inc.), subjects were rapidly switched between breathing room air and carbon dioxide, and back to room air. All four carbon dioxide administrations consisted of one breath each. Peripheral chemosensitivity to hypercapnia was calculated by plotting the mean of the three highest consecutive ventilations (e.g., individual breaths extrapolated to minute values) versus the maximum PETCO_2_ value within 2 min following each carbon dioxide administration (Chua and Coats 1995; Edelman et al. 1973; Pfoh et al. 2016). Furthermore, recent findings indicate that activation of the peripheral chemoreceptors also modulate hemodynamics (Stickland et al. 2007, 2008; Niewinski et al. 2014a; Limberg et al. 2015). Therefore, peripheral chemosensitivity to hypercapnia was also calculated by plotting the peak heart rate and the peak mean arterial pressure versus the maximum PETCO_2_ value within 2 min following each carbon dioxide administration, using similar methods that have been used during acute hypoxia (Niewinski et al. 2013, 2014a,b; Limberg et al. 2016). Peripheral chemosensitivity to hypercapnia data are reported as the slope of the linear regression line for the ventilatory, heart rate, and blood pressure responses to hypercapnia. This test of peripheral chemosensitivity is reliable and reproducible within subjects over 1 month (Chua and Coats 1995).

### Peripheral chemosensitivity to hypoxia

Peripheral chemosensitivity to hypoxia was measured via four nitrogen administrations (i.e., 100% N_2_) separated by 3 min of room air breathing. Briefly, using a pneumatic switching valve (Hans Rudolph, Inc.), subjects were rapidly switched between breathing room air and nitrogen, and back to room air. The first two nitrogen administrations consisted of two and four breaths, respectively, for all subjects. The number of nitrogen breaths for each of the remaining two nitrogen administrations were determined based on the SpO_2_ values achieved during the first two nitrogen administrations, and kept consistent within a subject during each peripheral chemosensitivity test for both experimental visits. Our goal was to achieve a range of nadir SpO_2_ values (80–95%) following the nitrogen administrations. Peripheral chemosensitivity to hypoxia was calculated by plotting the mean of the three highest consecutive ventilations (e.g., individual breaths extrapolated to minute values) versus the nadir SpO_2_ value within 2 min following each nitrogen administration (Edelman et al. 1973; Weil and Zwillich 1976; Chua and Coats 1995; Niewinski et al. 2013, 2014a,b; Limberg et al. 2015; Pfoh et al. 2016). Peripheral chemosensitivity to hypoxia was also calculated by plotting the peak heart rate and the peak mean arterial pressure versus the nadir SpO_2_ value within 2 min following each nitrogen administration (Edelman et al. 1973; Chua and Coats 1995; Niewinski et al. 2013, 2014a,b; Limberg et al. 2016). Peripheral chemosensitivity to hypoxia data are reported as the absolute value of the slope of the linear regression line for the ventilatory, heart rate, and blood pressure responses to hypoxia. This test of peripheral chemosensitivity was chosen to avoid ventilatory decline that is associated with longer hypoxic durations (Powell et al. 1998; Steinback and Poulin 2007; Pfoh et al. 2016). This test of peripheral chemosensitivity is also reliable and reproducible within subjects over 1 month (Chua and Coats 1995).

### Data and statistical analyses

Resting data were determined using the mean values from the last 2 min of each seated rest period, prior to the tests of peripheral chemosensitivity. Data were assessed for approximation to a normal distribution and sphericity, and no corrections were necessary. Outliers were identified and removed using a nonlinear regression analysis using the ROUT method in Prism (Motulsky and Brown 2006). The *Q* value, or the false discovery rate, was set conservatively (i.e., 0.1%) so that only definitive outliers were removed and the *n* is reported for each result. Objectively determined outliers were removed from statistical analyses for the ventilatory responses to hypercapnia and hypoxia (*n* = 2) and for the blood pressure responses to hypercapnia and hypoxia (*n* = 1). All data were analyzed using a two‐way repeated measures ANOVA. If a significant interaction or main effect was found, the Holm–Sidak multiple comparisons post hoc test was used to determine where differences existed. Data were compared to baseline within each visit and between visits at five time points (i.e., baseline, 10, 60, 120 min, and post). Data were analyzed using Prism software (Version 6; GraphPad Software Inc., La Jolla, CA). Data are reported as means ± SD and exact *P*‐values are reported where possible.

## Results

### Body weight and urine loss

Reductions in body weight were greater following HOWI versus control (−0.7 ± 0.5 kg vs. −0.2 ± 0.2 kg, *P* = 0.003). The greater reduction in body weight during HOWI can be mostly attributed to urine loss (HOWI: 0.7 ± 0.5 kg, Control: 0.1 ± 0.2 kg, *P* = 0.001).

### Ventilation

**Figure 1:**
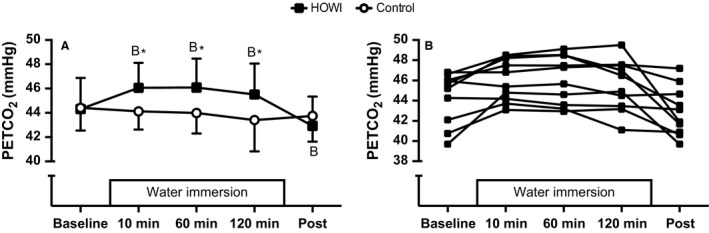
Mean values during HOWI and control (A) and individual values during HOWI (B) for PETCO_2_ at baseline, 10 min, 60 min, 120 min, and post. Values are mean ± SD,* n* = 10. *different from control, *P* < 0.050. B‐different from baseline, *P* < 0.050. HOWI, head out water immersion.

Mean values during HOWI and control (Fig. 1A) and individual values during HOWI (Fig. 1B) for PETCO_2_ are presented in Figure 1. PETCO_2_ was not statistically different during HOWI versus control at baseline (*P* = 0.840) or post (*P* = 0.142), but was higher during HOWI versus control at 10 min (46 ± 2 vs. 44 ± 2 mmHg, *P* < 0.001), 60 min (46 ± 2 vs. 44 ± 2 mmHg, *P* < 0.001), and 120 min (46 ± 3 vs. 43 ± 3 mmHg, *P* < 0.001). Furthermore, PETCO_2_ was higher at 10 min (*P* = 0.001), 60 min (*P* = 0.001), and 120 min (*P* = 0.011) versus baseline, and lower at post versus baseline (*P* = 0.007) during HOWI.

**Figure 2:**
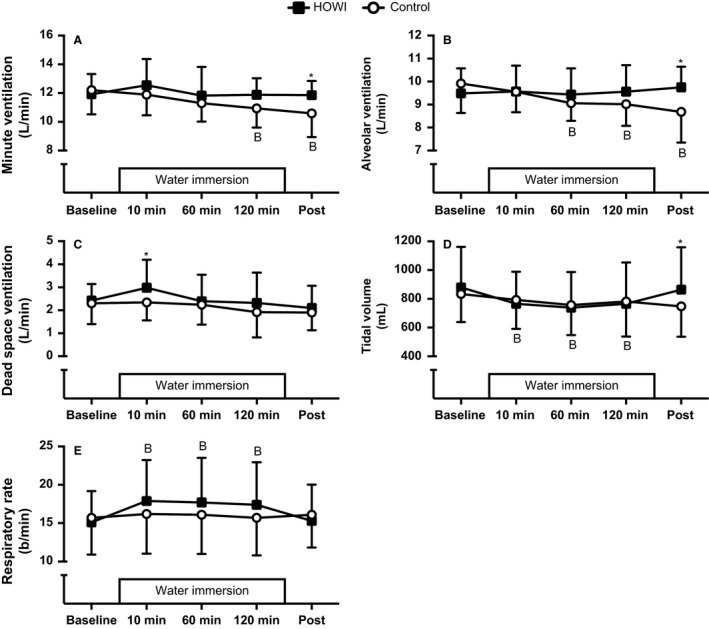
Minute ventilation (A), alveolar ventilation (B), dead space ventilation (C), tidal volume (D), and respiratory rate (E) at baseline, 10 min, 60 min, 120 min, and post HOWI and control. Values are mean ± SD,* n* = 10. *different from control, *P* < 0.050. B‐different from baseline, *P* < 0.050. HOWI, head out water immersion.

Minute ventilation (Fig. 2A) was not statistically different during HOWI versus control at baseline (*P* = 0.508), 10 min (*P* = 0.384), 60 min (*P* = 0.435), or 120 min (*P* = 0.156), but was higher during HOWI versus control at post (11.9 ± 1.0 vs. 10.6 ± 1.6 L/min, *P* = 0.037). Furthermore, ventilation was lower at 120 min (*P* = 0.021) and post (*P* = 0.003) versus baseline during control.

Alveolar ventilation (Fig. 2B) was not statistically different during HOWI versus control at baseline (*P* = 0.460), 10 min (*P* = 0.960), 60 min (*P* = 0.460), or 120 min (*P* = 0.315), but was higher during HOWI versus control at post (*P* = 0.008). Alveolar ventilation was lower at 60 min (*P* = 0.021), 120 min (*P* = 0.021), and post (*P* = 0.002) versus baseline during control.

Dead space ventilation (Fig. 2C) was not statistically different during HOWI versus control at baseline (*P* = 0.754), 60 min (*P* = 0.754), 120 min (*P* = 0.279), or post (*P* = 0.747), but was greater during HOWI versus control at 10 min (*P* = 0.029). Dead space ventilation was not statistically different versus baseline at any time point in either condition (*P* ≥ 0.061).

Tidal volume (Fig. 2D) was not statistically different during HOWI versus control at baseline (*P* = 0.636), 10 min (*P* = 0.836), 60 min (*P* = 0.859), or 120 min (*P* = 0.859), but was higher during HOWI versus control at post (*P* = 0.015). However, tidal volume was lower at 10 min (765 ± 222 mL, *P* = 0.011), 60 min (739 ± 249 mL, *P* = 0.002), and 120 min (765 ± 287 mL, *P* = 0.011) versus baseline (879 ± 282 mL) during HOWI.

Respiratory rate (Fig. 2E) was not statistically different during HOWI versus control at baseline (*P* = 0.461), 10 min (*P* = 0.103), 60 min (*P* = 0.103), 120 min (*P* = 0.103), or post (*P* = 0.461). However, respiratory rate was higher at 10 min (18 ± 5 b/min, *P* = 0.001), 60 min (18 ± 6 b/min, *P* = 0.002), and 120 min (17 ± 6 b/min, *P* = 0.005) versus baseline (15 ± 4 b/min) during HOWI.

### Hemodynamics

**Figure 3:**
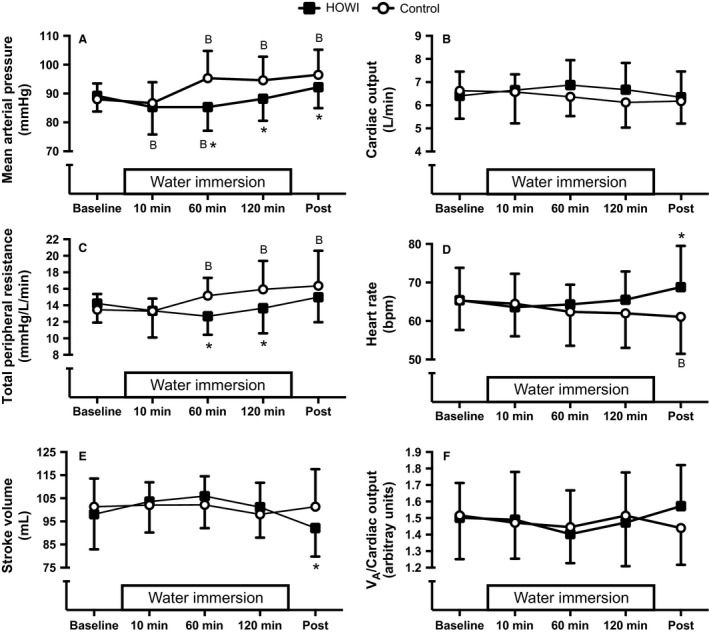
Mean arterial pressure (A), cardiac output (B), total peripheral resistance (C), heart rate (D), stroke volume (E), and alveolar ventilation (*V*_A_) to cardiac output ratio (F) at baseline, 10 min, 60 min, 120 min, and post HOWI and control. Values are mean ± SD,* n* = 10. *different from control, *P* < 0.050. B‐different from baseline, *P* < 0.050. HOWI, head out water immersion.

Mean arterial pressure (Fig. 3A) was not statistically different during HOWI versus control at baseline (*P* = 0.563) or 10 min (*P* = 0.563), but was lower during HOWI versus control at 60 min (85 ± 8 vs. 95 ± 9 mmHg, *P* < 0.001), 120 min (88 ± 8 vs. 95 ± 8 mmHg, *P* < 0.001), and post (92 ± 7 vs. 97 ± 9 mmHg, *P* = 0.016). Mean arterial pressure was lower at 10 min (*P* = 0.042) and 60 min (*P* = 0.042) versus baseline during HOWI. Mean arterial pressure was higher at 60 min (*P* < 0.001), 120 min (*P* < 0.001), and post (*P* < 0.001) vs. baseline during control.

Cardiac output (Fig. 3B) was not statistically different during HOWI versus control at any time point (condition main effect: *P* = 0.252). Moreover, cardiac output was not statistically different versus baseline at any time point in either condition (time main effect: *P* = 0.152).

Total peripheral resistance (Fig. 3C) was not statistically different during HOWI versus control at baseline (*P* = 0.431), 10 min (*P* = 0.969), or post (*P* = 0.095), but was lower during HOWI versus control at 60 min (*P* = 0.002) and 120 min (*P* = 0.003). Total peripheral resistance was not statistically different versus baseline at any time point during HOWI but was higher at 60 min (*P* = 0.021), 120 min (*P* = 0.001), and post (*P* < 0.001) versus baseline during control.

Heart rate (Fig. 3D) was not statistically different during HOWI versus control at baseline (*P* = 0.949), 10 min (*P* = 0.812), 60 min (*P* = 0.544), or 120 min (*P* = 0.117), but was higher during HOWI versus control at post (69 ± 11 vs. 61 ± 10 bpm, respectively, *P* < 0.001). During the control visit, heart rate was lower at post versus baseline (65 ± 8 bpm, *P* = 0.041).

Stroke volume (Fig. 3E) was not statistically different during HOWI versus control at baseline (*P* = 0.697), 10 min (*P *= 0.697), 60 min (*P* = 0.697), and 120 min (*P* = 0.697), but was lower during HOWI versus control at post (*P* = 0.037). Stroke volume was not statistically different versus baseline at any time point in either condition (*P* ≥ 0.085).

The alveolar ventilation to perfusion ratio (Fig. 3F) was not statistically different during HOWI versus control at any time point (condition main effect: *P* = 0.820). Moreover, the alveolar ventilation to perfusion ratio was not statistically different versus baseline at any time point in either condition (time main effect: *P* = 0.456).

### Peripheral chemosensitivity to hypercapnia

**Figure 4:**
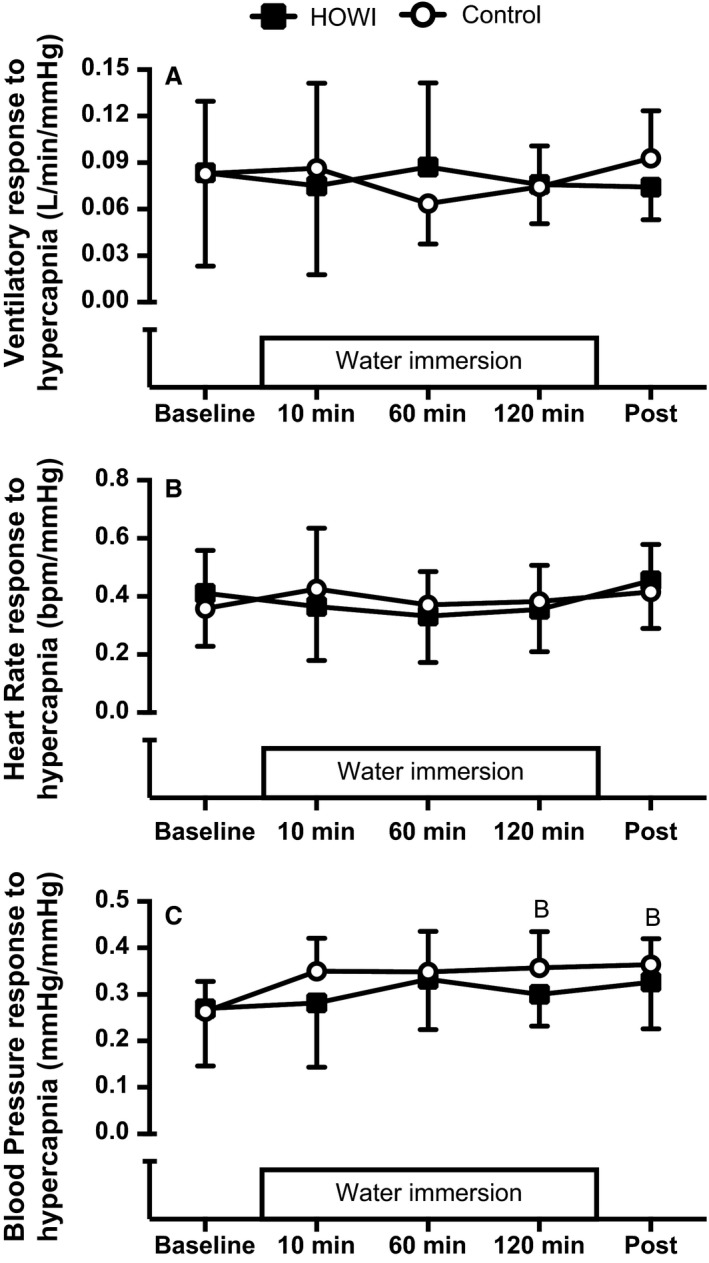
Ventilatory response to hypercapnia (A; *n* = 8), heart rate response to hypercapnia (B; *n* = 10), and blood pressure response to hypercapnia (C; *n* = 9) at baseline, 10 min, 60 min, 120 min, and post HOWI or control. Values are mean ± SD. B‐different from baseline, *P* < 0.050. HOWI, head out water immersion.

Ventilatory responses to hypercapnia (Fig. 4A) were not statistically different during HOWI versus control at any time point (condition main effect: *P* = 0.898). Moreover, ventilatory responses to hypercapnia were not statistically different versus baseline at any time point in either condition (time main effect: *P* = 0.951).

Heart rate responses to hypercapnia (Fig. 4B) were not statistically different during HOWI versus control at any time point (condition main effect: *P* = 0.760). Moreover, heart rate responses to hypercapnia were not statistically different versus baseline at any time point in either condition (time main effect: *P* = 0.339).

Mean arterial pressure responses to hypercapnia (Fig. 4C) were not statistically different during HOWI versus control at any time point (condition main effect: *P* = 0.092). However, mean arterial pressure responses to hypercapnia were higher at 120 min (*P* = 0.049) and post (*P* = 0.043) versus baseline during control.

Maximum PETCO_2_ during peripheral chemosensitivity to hypercapnia are presented in Table 1. Maximum PETCO_2_ was not statistically different during HOWI versus control at any time point (condition main effect: *P* = 0.398). Maximum PETCO_2_ was not statistically different versus baseline at any time point in either condition (time main effect: *P* = 0.789).

**Table 1:** Maximum PETCO_2_ and nadir SpO_2_ during peripheral chemosensitivity to hypercapnia and hypoxia tests

	Max PETCO_2_ (mmHg)		Nadir SpO^2^ (%)	
	HOWI	Control	HOWI	Control
Baseline	95 ± 6	94 ± 8	80 ± 6	83 ± 6
10 min	96 ± 6	94 ± 10	81 ± 7	82 ± 8
60 min	96 ± 5	96 ± 3	76 ± 7^a^	83 ± 6
120 min	95 ± 5	95 ± 5	79 ± 4^a^	85 ± 6
Post	95 ± 4	95 ± 5	81 ± 6	84 ± 6

Values are mean ± SD, *n* = 10.

a Different from control, *P* < 0.050.

### Peripheral chemosensitivity to hypoxia

**Figure 5:**
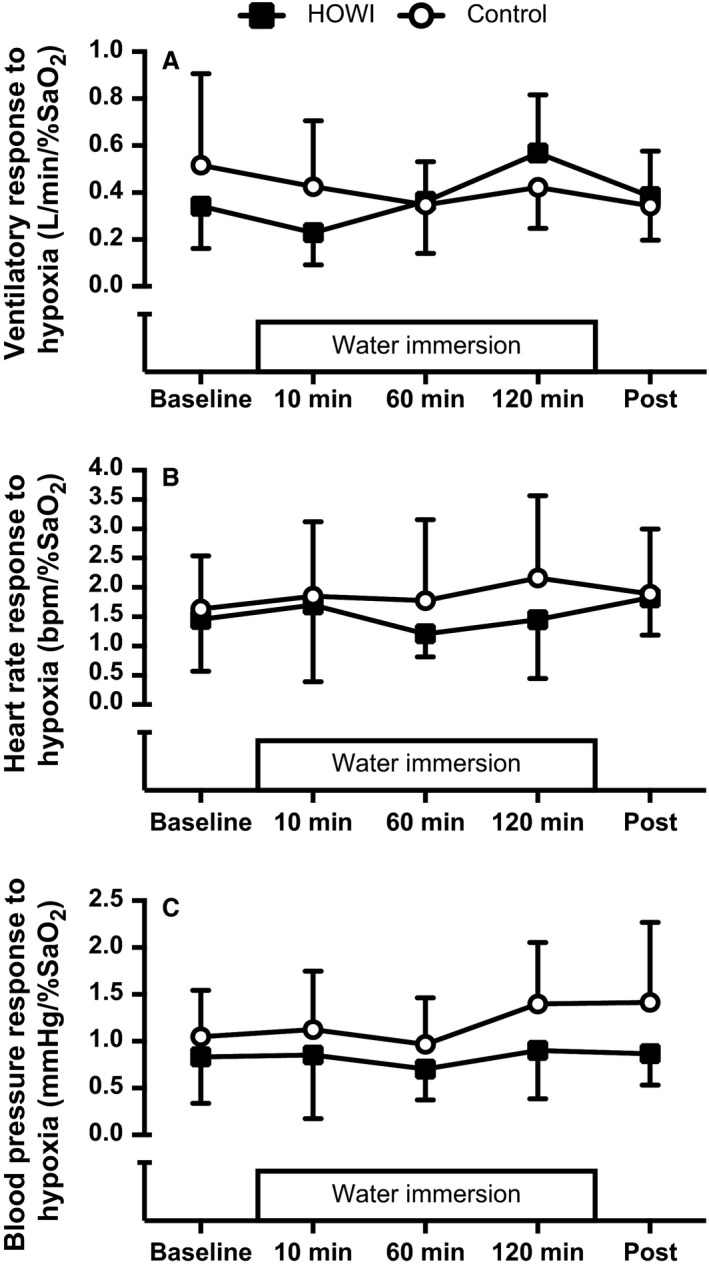
Ventilatory response to hypoxia (A; *n* = 8), heart rate response to hypoxia (B; *n* = 10), and blood pressure response to hypoxia (C; *n* = 9) at baseline, 10 min, 60 min, 120 min, and post, head out water immersion or control. Values are mean ± SD.

Ventilatory responses to hypoxia (Fig. 5A) were not statistically different during HOWI versus control at any time point (condition main effect: *P* = 0.714). Moreover, ventilatory responses to hypoxia were not statistically different versus baseline at any time point in either condition (time main effect: *P* = 0.099).

Heart rate responses to hypoxia (Fig. 5B) were not statistically different during HOWI versus control at any time point (condition main effect: *P* = 0.258). Moreover, heart rate responses to hypoxia were not statistically different versus baseline at any time point in either condition (time main effect: *P* = 0.235).

Mean arterial pressure responses to hypoxia (Fig. 5C) were not statistically different during HOWI versus control at any time point (condition main effect: *P* = 0.051). Moreover, mean arterial pressure responses to hypoxia were not statistically different versus baseline at any time point in either condition (time main effect: *P* = 0.246).

Nadir SpO_2_ during peripheral chemosensitivity to hypoxia are presented in Table 1. Nadir SpO_2_ was not statistically different during HOWI versus control at baseline (*P* = 0.367), 10 min (*P *= 0.440), or post (*P* = 0.340), but was lower during HOWI versus control at 60 min (*P* =0.010) and 120 min (*P* = 0.042). Nadir SpO_2_ was not statistically different versus baseline at any time point in either condition (time main effect: *P* = 0.135).

## Discussion

Our study demonstrates that PETCO_2_ increases during 2 h of thermoneutral HOWI in humans without a change in ventilation or peripheral chemosensitivity (Figs 1, 4, and 5). Contrary to our hypothesis, peripheral chemosensitivity to hypercapnia and hypoxia was not blunted during HOWI (Figs. 4 and 5). Collectively, these data indicate that activation of the peripheral chemoreceptors to a brief hypercapnic or hypoxic stimulus is not altered during HOWI. Consequently, our data do not support a role for the peripheral chemoreceptors in the retention of CO_2_ during thermoneutral HOWI in humans.

### Ventilation

Similar to previous findings (Jarrett 1966; Salzano et al. 1970, 1984; Kerem et al. 1995; Cherry et al. 2009; Miyamoto et al. 2014), we observed a significant increase in PETCO_2_ during HOWI (Fig. 1A). It has been shown that CO_2_ retention occurs during water immersion at depth due a reduction in alveolar ventilation that is caused by increased dead space (Salzano et al. 1984; Mummery et al. 2003). However, our subjects were studied at the surface (i.e., 1 ATA) and therefore the increase in dead space in our subjects was most likely lower compared to subjects that have been studied at depth (Salzano et al. 1984; Hickey et al. 1987; Mummery et al. 2003; Cherry et al. 2009). The breath by breath ventilatory data from our study indicate that ventilation was not altered throughout HOWI. In addition to an increase in dead space, it has been suggested that an increase in PETCO_2_ may be due to an increase in CO_2_ redistribution and storage throughout body tissues (Farhi and Rahn 1960; Matalon and Farhi 1979; Serrador et al. 1998). It is unclear if CO_2_ redistribution and storage occurred during our study. Recent evidence indicates that thermoneutral HOWI shifts the respiratory operating point (i.e., PETCO_2_ vs. minute ventilation) to the right to increase the likelihood of CO_2_ retention (Miyamoto et al. 2014). Our data agree with the idea that thermoneutral HOWI shifts the respiratory operating point as we observed an increase in PETCO_2_ without a change in ventilation.

Previous findings indicate that minute ventilation and alveolar ventilation are reduced during water immersion, primarily as a function of increased breathing gas density (Salzano et al. 1984; Cherry et al. 2009). It is also thought that central hypervolemia and increased work of breathing during water immersion contribute to the reductions in minute and alveolar ventilation (Lanphier and Bookspan 1999; Lundgren and Miller 1999). Our data (Fig 2A and B) do not confirm the reductions in minute and alveolar ventilation. However, we did observe an increase in dead space ventilation at 10 min of HOWI which is similar to other investigations (Mummery et al. 2003; Cherry et al. 2009). Thus, the CO_2_ retention that we observed during water immersion might be related to the increased dead space and not a reduction in alveolar ventilation. This idea warrants future investigation.

Changes in breathing pattern might also contribute to the increased CO_2_ retention during water immersion. We observed decreases in tidal volume and increases in respiratory rate throughout HOWI compared to baseline (Fig. 2D and E). Water immersion has been shown to increase the work of breathing (Otis et al. 1950; Collett and Engel 1986) but previous studies suggest that this is not directly related to CO_2_ retention (Thalmann et al. 1979; Hickey et al. 1987; Norfleet et al. 1987). Thus, the changes in breathing pattern that we observed, possibly due to the enhanced negative pressure breathing (Pendergast and Lundgren 2009), could be responsible for CO_2_ retention during HOWI. However, it is unknown if the increased work of breathing is mitigated via alterations in breathing pattern (Cherry et al. 2009). A reduced alveolar ventilation, which is proposed to be the one of the main causes of CO_2_ retention (Salzano et al. 1984; Mummery et al. 2003), is thought to occur in place of increasing the work of breathing to prevent hypercapnia during water immersion (Lundgren and Miller 1999). On the basis of our alveolar ventilation and dead space data, we speculate that HOWI may induce alterations in breathing pattern to minimize the work of breathing which subsequently leads to CO_2_ retention.

### Hemodynamics

The prevailing theory is that mean arterial pressure initially increases during water immersion due to a cephalad fluid shift which subsequently causes diuresis and a return of blood pressure to baseline values after continued water immersion (Arborelius et al. 1972; Pendergast et al. 2015). However, some investigators have also found that mean arterial pressure does not change (Bonde‐Petersen et al. 1992; Sramek et al. 2000; Watenpaugh et al. 2000; Pendergast et al. 2015) or slightly decreases (Craig and Dvorak 1966). We observed a decrease in mean arterial pressure at 10 min and 60 min of HOWI compared to baseline (Fig. 3A), which could be explained by a decrease in total peripheral resistance (Fig. 3C) (Arborelius et al. 1972; Bonde‐Petersen et al. 1992; Pendergast et al. 2015) and/or diuresis without a change in cardiac output (Fig. 3B). The water temperature we used (~35°C) (Pendergast et al. 2015) may have slightly heated the integument due to the water temperature to skin temperature (~33–34°C) thermal gradient (Bierman 1936), which may have increased intersubject variability in total peripheral resistance.

It is thought that inequality of the alveolar ventilation to perfusion ratio (i.e., <1) occurs during diving as a function of the reduced alveolar ventilation and the increased blood flow. However, previous findings indicate that the alveolar ventilation to perfusion ratio is unaffected during thermoneutral HOWI (Derion et al. 1992). Our data agree with the findings of Derion et al., and can be explained by the fact that we did not observe a reduced alveolar ventilation and/or an increased cardiac output during water immersion. Thus, we suggest that alveolar ventilation to perfusion mismatching does not occur during water immersion and does not contribute to the explanation of CO_2_ retention.

### Peripheral chemosensitivity to hypercapnia

Our data indicate that ventilatory and hemodynamic responses to acute hypercapnia are not blunted during 2 h of thermoneutral HOWI (Fig. 4A). Therefore, it appears as though CO_2_ retention during HOWI is not due to a reduction in the sensitivity of the peripheral chemoreceptors to a brief hypercapnic stimulus. Furthermore, there is an interaction between the central and peripheral chemoreceptors such that the ventilatory response to central chemoreceptor stimulation is reliant upon activation of the peripheral chemoreceptors (Rodman et al. 2001; Smith et al. 2006, 2015; Blain et al. 2010). Based on our findings that the ventilatory response to hypercapnia is not blunted during 2 h of thermoneutral HOWI, it is likely that central chemosensitivity is also not changed. However, it is not known if central chemosensitivity is altered beyond 10 min of thermoneutral HOWI (Chang and Lundgren 1995).

### Peripheral chemosensitivity to hypoxia

Similar to the peripheral chemosensitivity to hypercapnia, we found that the ventilatory and hemodynamic responses to acute hypoxia are not blunted during HOWI (Fig. 5A). In support of our findings, the use of lower body positive pressure to increase central blood volume does not alter the ventilatory response to hypoxia (Koehle et al. 2010). However, Heistad et al. (1975) demonstrated that baroreflex loading lowers the ventilatory response to peripheral chemoreceptor activation. Thermoneutral HOWI induces central hypervolemia of ~1 L (Arborelius et al. 1972), which should be sufficient to load the arterial baroreceptors (Pendergast et al. 2015). However, we did not observe an increase in mean arterial pressure during HOWI. Therefore, we might not have sufficiently loaded the baroreceptors to cause a decrease in peripheral chemosensitivity during HOWI (Heistad et al. 1975). It is currently not known if further activation of the sympathetic nervous system modulates peripheral chemosensitivity during HOWI as circulating catecholamines have been shown to be important modulators of peripheral chemosensitivity (Prabhakar and Peng 2004; Stickland et al. 2007, 2008; Niewinski et al. 2014b) and there is evidence that demonstrates that circulating catecholamines are lower during thermoneutral HOWI (Norsk et al. 1990; Stadeager et al. 1992).

### Perspectives

Although the degree of CO_2_ retention induced from 2 h of resting thermoneutral HOWI is not large enough to develop CO_2_ narcosis, CO_2_ retention merits formal investigation because of the likelihood of CO_2_ narcosis during diving (Warkander et al. 1990; Lanphier and Bookspan 1999). Our data indicate that peripheral chemosensitivity is not changed and it does not appear that the peripheral chemoreceptors contribute to CO_2_ retention during 2 h of thermoneutral HOWI. Moreover, Chang & Lundgren have previously shown that the central chemosensitivity is not altered during 10 min of thermoneutral HOWI and most likely do not contribute to CO_2_ retention. However, Cherry and colleagues have shown that CO_2_ retention occurs in a graded response to multiple factors including increased gas density and breathing resistance, as well as minor factors such as baseline central chemosensitivity and baseline aerobic fitness (i.e., maximal oxygen consumption) (Cherry et al. 2009). Furthermore, they also showed that greater decreases in ventilation lead to greater CO_2_ retention (Cherry et al. 2009). However, we showed that CO_2_ retention may occur independent of any changes in ventilation. Thus, it is important to further evaluate other possible mechanisms that contribute to the degree of CO_2_ retention during HOWI (i.e., central chemosensitivity, hyperoxia, breathing resistance, immersion depth, and oxygen consumption).

### Considerations

Our study has several limitations. First, the tests of peripheral chemosensitivity were not randomized. Throughout the protocol, subjects always experienced four nitrogen administrations followed by four carbon dioxide administrations. However, it has previously been shown that repetitive hypoxic administrations do not induce long‐term facilitation of ventilation in humans (McEvoy et al. 1996; Powell et al. 1998). In spite of our efforts to blind subjects to the gas administrations (i.e., timing and content), they were most likely aware of when they inhaled the hypercapnic gas due to the acidic taste and subjects were able to see and/or hear the pneumatic switching valve. Consequently, it is possible that subjects altered their ventilation upon administration of the hypoxic or hypercapnic gases. However, we believe that this effect was minimized by familiarizing the subjects with the gases and switching value prior to the experimental visits. Peripheral chemosensitivity to hypercapnia was achieved using only 1 breath of hypercapnic gas during each administration. Thus, our hypercapnic stimulus (i.e., maximum PETCO_2_) was similar following each gas administration and we did not obtain a range of maximum PETCO_2_ values, similar to how we obtained a range of nadir SpO_2_ during the hypoxic administrations. It is unclear if ventilatory responses to acute hypercapnia are linear throughout a wide range of maximum PETCO_2_. The nadir SpO_2_ during peripheral chemosensitivity to hypoxia indicate that the hypoxic stimulus was greater at 60 min and 120 min of HOWI versus control (Table 1). However, our calculation of peripheral chemosensitivity is based on a linear relationship between SpO_2_ and minute ventilation, which is linear until SpO_2_ falls below 70% (Chua and Coats 1995). Finally, CO_2_ retention occurred during HOWI. Therefore, during the HOWI visit, the tests of peripheral chemosensitivity took place with a mild hypercapnic background which may have activated the central chemoreceptors and potentially masked changes in peripheral chemosensitivity (Somers et al. 1989; Smith et al. 2006; Blain et al. 2010). However, because ventilation was unchanged throughout HOWI, we speculate that this did not contribute to our findings.

## Conclusions

In summary, 2 h of thermoneutral HOWI caused an increase in PETCO_2_, which indicates that subjects experienced CO_2_ retention, without a decrease in ventilation. Additionally, peripheral chemosensitivity to acute hypercapnia and acute hypoxia was not blunted during HOWI. Therefore, the sensitivity of the peripheral chemoreceptors to hypercapnia and hypoxia does not appear to contribute to the increase in CO_2_ retention during 2 h of thermoneutral HOWI in healthy young adults.

## Conflict of Interest

No conflicts of interest, financial or otherwise, are declared by the authors.
